# A phased chromosome-level genome of the annelid tubeworm *Galeolaria caespitosa*

**DOI:** 10.1093/jhered/esaf025

**Published:** 2025-04-22

**Authors:** Monique van Dorssen, Emily K Belcher, Cristóbal Gallegos, Kathryn A Hodgins, Keyne Monro

**Affiliations:** School of Biological Sciences, Monash University, Victoria, Australia; School of Biological Sciences, Monash University, Victoria, Australia; School of Biological Sciences, Monash University, Victoria, Australia; School of Biological Sciences, Monash University, Victoria, Australia; School of Biological Sciences, Monash University, Victoria, Australia

**Keywords:** Annelida, annotated reference genome, ecosystem engineer, marine invertebrate, mitochondria, Polychaeta

## Abstract

Haplotype-resolved (phased) genome assemblies are emerging as important assets for genomic studies of species with high heterozygosity, but remain lacking for key animal lineages. Here, we use PacBio HiFi and Omni-C technologies to assemble the first phased, annotated, chromosome-level genome for any annelid: the reef-building tubeworm *Galeolaria caespitosa* (Serpulidae). The assembly is 803.5 Mbp long (scaffold N50 = 76.5 Mbp) for haplotype 1 and 789.3 Mbp long (scaffold N50 = 75.4 Mbp) for haplotype 2, which are arranged into 11 pairs of chromosomes showing no sign of sex chromosomes. This compares with cytological analyses reporting 12 to 13 pairs in *G. caespitosa*’s closest relatives, including species that are protandrous hermaphrodites. We combined long-read and short-read transcriptome sequencing to annotate both haplotypes, resulting in 30,495 predicted proteins for haplotype 1, 27,423 proteins for haplotype two, and 79.5% of proteins with at least one functional annotation. We also assembled a mitochondrial genome 23 kbp long, annotating all genes typically found in mitochondrial DNA apart from those coding the *16S* ribosomal subunit (*rrnL*) and the protein *atp8—*a short, fast-evolving mitochondrial gene missing in other metazoans. Comparing *G. caespitosa*’s genome to those of three other annelids reveals limited collinearity despite 36.0% of shared orthologous gene clusters (4,238 of 11,763 clusters counted in *G. caespitosa*), suggesting extensive chromosomal rearrangements among lineages. New high-quality annelid genomes may help resolve the genetic and evolutionary basis of this diversity.

## Introduction

Genomic tools are increasingly used to understand biodiversity, but remain out of reach for most species of eco-evolutionary or conservation concern due to a lack of high-quality reference genomes (Formenti et al. 2022; Paez et al. 2022). Such genomes greatly enhance genomic insights by providing templates for mapping genomic information and studying its broader function (Hoban et al. 2016; Mérot et al. 2020). To date, however, most genome assemblies remain in draft form (i.e. fragmented and unannotated), or merge the parental haplotypes present in the sequenced individual (Duitama 2023). Although chromosome-level assemblies are fast accumulating, haplotype-merged ones may only be accurate for inbred model species, or species with low heterozygosity, where haplotypes are genetically similar (Takeuchi et al. 2022). For outbred, highly heterozygous species, merging haplotypes may hide substantial variation between them and distort our view of genetic diversity.

Marine invertebrates, and broadcast spawners especially, are known for high levels of heterozygosity owing to large population sizes, high fecundities, and extensive dispersal of gametes, embryos, and larvae (Plough 2016; Lotterhos et al. 2021). Moreover, by representing the majority of extant animal phyla and including the oldest metazoans, marine invertebrate genomes may encode not only unique gene products, but also key insights about adaptation and diversification (Lopez et al. 2019). Genome assemblies that are phased into haplotypes, and capture information about heterozygosity, are therefore essential to unlock and study genetic diversity in this group. High heterozygosity brings challenges to such assemblies, since the presence of divergent alleles can cause assembly fragmentation or errors, but advances in long-read sequencing technologies now enable phasing in the absence of parental sequencing or pedigree data (Cheng et al. 2021, 2022). Currently, though, only a handful of phased or well-annotated genomes exist for marine invertebrates, covering only a small sample of phyla such as mollusks (Takeuchi et al. 2022) and echinoderms (Schiebelhut et al. 2023).

Annelids remain one of the most diverse, yet poorly understood, metazoan phyla (Sun et al. 2021). They have evolved a remarkable array of reproductive and developmental modes (Dorresteijn and Westheide 1999), and shape the functioning of benthic communities from shallow waters to deep-sea trenches (Hutchings 1998). Annelid genomes could thus provide major insights into life-history evolution and adaptation to environmental change. Despite the biodiversity captured in this group, however, we know surprisingly little about the genomic basis of annelid life histories or their diversification (Lopez et al. 2019; Sun et al. 2021). Of the 48 chromosome-level genome assemblies currently available for annelids on the National Center for Biotechnology Information (NCBI) (accessed February 2025), few are published (but see Jin et al. 2020; Zakas et al. 2022), and none appear to be both phased and annotated. Such oversight leaves major gaps in our understanding of basic evolutionary biology and genome evolution in a key metazoan lineage, and limited tools for progress on this front.

Here, we present the first phased and annotated chromosome-level genome for any annelid—the reef-building tubeworm, *Galeolaria caespitosa*. The species is a broadcast-spawner endemic to rocky shores of temperate Australia, where it is an ecosystem engineer (Wright and Gribben 2017; Cole et al. 2018) and bioindicator of ecosystem health (Ross and Bidwell 2001; Lu et al. 2017). The species also belongs to the Serpulidae, an ecologically important family that includes notorious biofoulers and bioinvaders (Sun et al. 2021), and life histories spanning diverse modes of sex determination, reproduction, and development (Kupriyanova et al. 2001). Little is known about the genomic basis of these patterns, despite them coinciding with changes in chromosome number (Dasgupta and Austin 1960), structural rearrangements within chromosomes (Dixon et al. 1998), and accelerated substitution rates and structural rearrangements within mitochondrial genomes (Sun et al. 2021). As the first annelid genome of this quality, the *G. caespitosa* genome will be valuable in its own right and serve as a reference for comparison with other individuals, species, and metazoan lineages.

## Methods

### DNA extraction, library preparation, and sequencing

We assembled the genome of a wild male sampled from Chelsea Pier (Victoria, Australia). The male was removed from its tube to induce spawning, then sperm and muscle tissue were frozen separately in liquid nitrogen and stored at −80 °C until DNA extraction.

High-molecular-weight genomic DNA was extracted from sperm using Qiagen’s Blood & Cell Culture DNA Kit. Yield and mean fragment size were assessed using a Qubit fluorometer and pulsed-field gel electrophoresis, respectively, and purity was confirmed using 260/280 and 260/230 absorbance ratios on a NanoDrop spectrophotometer. A PacBio HiFi library was prepared using PacBio’s SMRTbell Express Template Prep Kit and sequenced in two SMRT Cells on the PacBio Sequel II platform (Cantata Bio, California, USA).

A Dovetail Omni-C library was prepared from muscle tissue using Dovetail’s Omni-C Kit. Chromatin was fixed in place in the nucleus using formaldehyde before digestion with DNase I. Chromatin ends were repaired and ligated to a biotinylated bridge adapter, followed by proximity ligation of adapter-containing ends and reversal of crosslinks. DNA was then purified and treated to remove biotin outside ligated fragments. The library was constructed using NEBNext Ultra enzymes and an Illumina-compatible adapter, with biotin-containing fragments isolated by streptavidin beads before Polymerase Chain Reaction (PCR) enrichment. Sequencing was done on the Illumina HiSeq X platform (Cantata Bio, California, USA).

### Initial scaffold-level assembly

All software used can be found in Table 1. From Omni-C reads, we estimated *k*-mer counts (*k *= 21) using *jellyfish* v2.3.0 (Marçais and Kingsford 2011), *k*-mer completeness using *Merqury* v1.3 (Rhie et al. 2020), and genome size and heterozygosity using *GenomeScope* v2.0 (Ranallo-Benavidez et al. 2020) with default settings (Supplementary Fig. S1A).

**Table 1.** Assembly software and pipeline.

Step	Software	Version
Genome assembly		
De novo assembly (contiging)	*HiFiasm*	0.15.4-r347
Scaffolding	*YaHS*	1.1
Linked density plot	*Juicebox*	1.9.8
*K*-mer counting	*jellyfish*	2.3.0
Estimation of genome size and heterozygosity	*GenomeScope*	2.0
Scaffold selection	*SciStatCalc k-means++*	2 October 2020
Genome–genome alignment	*minimap2*	2.17-r954
	*R*	4.3.0
Genome quality assessment		
Basic assembly metrics	*QUAST*	5.0.2
Assembly completeness	*BUSCO* with eukaryote gene set	5.1.3
	*Merqury*	1.3
Organelle assembly		
Mitogenome assembly	*MitoHiFi*	3.2.1
	*MITOS*	2.1.0
	*MitoFinder*	1.4.0
	*circularMT*	June 2024
Iso-Seq processing		
Primer removal	*lima*	2.9.0
Poly(A) trimming	*isoseq3*	4.0.0
RNA-Seq processing		
Filtering and adapter removal	*fastp*	0.20.0
Structural annotation		
Repeat modeling	*RepeatModeler2*	2.0.5
Protein excluding	*ProtExcluder*	1.2
Repeat masking	*RepeatMasker*	4.1.1
Long-read mapping	*minimap2*	2.17-r954
Short read mapping	*STAR*	2.7.11
	*edgeR*	4.2.1
SAM/BAM processing	*samtools*	1.9-24
Collapsing	*isoseq3*	4.0.0
Transcriptome assembly	*stringtie*	2.1.5
Genome annotation	*BRAKER*	3.0.7
	*GeneMarkS-T*	5.1
	*Tsebra*	1.1.2.5
Convert to gff3	*AGAT*	1.0.0
Clean output file	*GFFtk*	24.2.4
Extract protein sequences to fasta	*gffread*	0.12.7
Functional annotation		
	*InterProScan*	5.61.93
	*Eggnog-mapper*	2.1.10
	*funannotate*	1.8.14
	*Diamond-BLASTp*	1.19
	*gffutils*	0.13
Comparative analysis		
	*OrthoVenn3*	2022
	*BLAST+*	12.15.0
	*MCScanX*	0.8

Citations are listed in the text, and scripts are available at https://github.com/moniquevdor/GaleolariaReferenceGenome.

To obtain phased contigs (haplotype 1 and haplotype 2) from HiFi reads, we used *Hifiasm* v0.15.4-r347 (Cheng et al. 2021) in *Hi-C Integrated-Assembly* mode with default parameters and Omni-C reads for phasing. We also re-ran *Hifiasm* with the *--hom-cov* parameter adjusted to the homozygous read coverage estimated by *GenomeScope* (37). Since this produced a less contiguous assembly (increasing L90 from 73 to 113 for haplotype 1, and from 72 to 105 for haplotype 2), we retained the original one.

We scaffolded phased contigs using *YaHS* v1.1 and Omni-C reads (Zhou et al. 2022), then calculated genome summary statistics for each scaffold-level assembly using *QUAST* v5 (Mikheenko et al. 2018). We checked contiguity using chromatin contact maps (Supplementary Fig. S1B) made in *Juicebox* v1.9.8 (Durand et al. 2016), and checked completeness using *BUSCO* v5.1.3 (Simão et al. 2015) queried against the eukaryote database (*eukaryota_odb10*, created January 2024 with 70 genomes and 255 Benchmarking Universal Single-Copy Orthologs [BUSCOs]).

### Chromosome-level genome assembly

We identified chromosomes from chromatin contact maps, which assemble chromosome-level scaffolds by translating the proximity of genomic regions in 3D space to contiguous linear organization. To test if the number of chromosomes identified from maps corresponded to a drop in scaffold size, we performed a clustering analysis on the lengths of the 20 longest scaffolds using the *k-means++* method (Arthur & Vassilvitskii, 2007) implemented using *SciStatCalc* (https://scistatcalc.blogspot.com/). After selecting the 11 largest scaffolds as chromosomes, we aligned the two haplotypes using *minimap2* v2.16 (Li 2018), plotted alignments in R using *ggpubr* (Kassambara 2023) and *pafr* (Winter et al. 2022), and created dot plots from *paf*-files containing alignments (Supplementary Fig. S1C). This identified chromosomes that matched between haplotypes, letting us rename chromosomes accordingly. We then moved the largest version of each chromosome into haplotype 1 and the other version into haplotype 2, meaning chromosome-level haplotypes differ from scaffold-level ones above.

We recalculated genome summary statistics for chromosome-level assemblies with *QUAST*, *Merqury*, and *BUSCO*, as above.

### Mitochondrial genome assembly

We extracted an initial mitochondrial assembly from the scaffold-level haplotype 1 using *MitoHifi* (https://github.com/marcelauliano/MitoHiFi; Uliano-Silva et al. 2023), with *MITOS* (Bernt et al. 2013) as the annotation tool and the mitochondrial genome of the serpulid, *Hydroides elegans* (GenBank accession number NC_061743.1), as the starting reference. Since several expected genes were missing, a second annotation was performed with *MitoFinder* (Allio et al. 2020). We compared our genome with that of *H. elegans* and another serpulid, *Spirobranchus giganteus* (Seixas et al. 2017), by visualizing genomes using *circularMT* (Goodman and Carr 2024).

### Transcriptome sequencing for genome annotation

Both long-read and short-read transcriptomes were used for annotation. We generated full-length transcripts from pooled adults (also from Chelsea Pier) and developmental stages obtained by spawning and cross-fertilizing adults (Rebolledo et al. 2023; Gallegos et al. 2024). Developing embryos and larvae were sampled at appropriate times (24 hours to 12 days) and stored at −80 °C until RNA extraction. We generated short-read transcripts from 91 samples of embryos obtained by spawning and cross-fertilizing additional sets of pooled adults (from Brighton Pier, Victoria, Australia). Total RNA was extracted separately from adult males, adult females, eggs, embryos, early larvae, and advanced larvae. Extraction required optimization by life stage, but used TRIzol, a PureLink RNA Mini Kit with or without TRIzol, or a Qiagen RNeasy Plus Mini Kit with mercaptoethanol, following manufacturer instructions.

RNA for long-read transcripts was pooled across life stages, deployed in PacBio Iso-Seq library preparation using PacBio’s SMRTbell Express Template Prep Kit, and sequenced on the PacBio Sequel IIe platform (Azenta Life Sciences, GENEWIZ Genomics Centre, Suzhou, China). Primers were removed and poly(A) tails trimmed from transcripts using *lima* v2.9.0 and *isoseq3* v4.0.0, respectively, from PacBio’s *Iso-Seq* pipeline. RNA for short-read transcripts underwent Illumina Stranded mRNA library preparation with poly(A) enrichment and was sequenced on the NovaSeq X Plus platform (Australian Genome Research Facility, Melbourne, Australia). Adapters and low-quality sequences were removed using *fastp* v0.20.0 (Chen et al. 2018, Chen 2023).

### Structural annotation and gene models

Both chromosome-level haplotypes were structurally annotated, after softmasking repeats using *RepeatModeler2* (Flynn et al. 2020) and *RepeatMasker* v4.1.1(Smit et al. 2013-2015) to improve gene-model predictions. All known annelid proteins (151,839 in the UniProtKB database; The UniProt Consortium 2022) were excluded from repeats using *ProtExcluder* v1.2 (Campbell et al. 2013). Repeat modeling was also used to characterize repeat type and density for each haplotype.

To predict gene models and generate a structural annotation for each haplotype, we combined Iso-Seq and RNA-seq evidence with evidence from annelid proteins downloaded from UniProtKB. First, we mapped Iso-Seq reads to haplotypes using *minimap2* v2.17 (Li 2018), sorted and collapsed mapped reads using *samtools* v1.9 (Danecek et al. 2021) and *isoseq3* v4.0.0 (PacBio 2018), respectively, and assembled the transcriptome using *stringtie* v2.1.5 (Pertea et al. 2015). We also mapped RNA-seq reads to haplotypes using *STAR* v2.7.11 (Dobin et al. 2012). Next, following the BRAKER long-read integration protocol (Stanke et al. 2006, 2008; Hoff et al. 2019), we annotated genes using BRAKER1 (Hoff et al. 2016) on mapped RNA-seq reads, BRAKER2 (Brůna et al. 2021) on UniProtKB protein sequences, and GeneMarkS-T (Tang et al. 2015) on mapped Iso-seq reads. Last, we used TSEBRA (Gabriel et al. 2021) to combine the three gene sets based on all evidence. We used the long-read configuration with increased weights of short-read and long-read hint sources, as recommended when the protein database lacks close relatives, and a threshold for intron support of 0.2 (Gabriel et al. 2021). This produced a *gtf* file with structural annotations and gene models.

We converted the *gtf* file to a *gff3* file using AGAT v1.0.0 (Dainat 2023), cleaned the *gff3* file using *GFFtk* v24.2.4 (https://github.com/nextgenusfs/gfftk), then extracted protein sequences using *gffread* v0.12.7. We filtered out gene models that showed no or very low expression using *edgeR* v4.2.1 (*filterByExpr* function; Chen et al. 2025), unless they overlapped with mapped Iso-seq reads or contained a protein hit (see Functional annotation). To check the completeness of our predicted protein set, we used *BUSCO* v5.1.3 as above.

### Functional annotation

Chromosome-level haplotypes were also functionally annotated by adapting the pipeline of Santangelo et al. (2023; https://github.com/James-S-Santangelo). We added functional annotations by querying predicted proteins against the databases below, before merging and formatting annotations for upload to NCBI. First, we retrieved functional annotations using *InterProScan* v5.61.93-0 (Jones et al. 2014) and *Eggnog-mapper* v2.1.10 (Cantalapiedra et al. 2021). Outputs were passed to *funannotate* v1.8.14 (Palmer and Stajich 2019), which combined annotations and queried additional databases (*merops* v12.0, *uniprot* v2024-01, *dbCan* v12.0, *pfam* v 36.0, *repeats* v.1.0, *go* 2024-01-17, *mibig* v1.4, *interpro* v98.0, *busco_outgroups* v1.0 metazoa database, and *gene2product* v.1.92). Proteins annotated as hypothetical, but containing 4-digit Enzyme Commission (EC) numbers, had annotations replaced with EC number products in the *ExPASSY* Enzyme database (Bairoch 2000). We also BLASTed protein-coding gene models against the NCBI *nr* database using *Diamond-BLASTp* v1.19 (Buchfink et al. 2021), adding outputs to the final *gff3* file using *gffutils* v0.13. Last, we mapped Iso-Seq and RNA-seq reads to the genome using *minimap2* 217-r954 and *STAR* v2.7.11, respectively, and removed hypothetical proteins lacking any mapped reads. We checked the completeness of our final annotated protein set using *BUSCO* v5.1.3 as above.

### Comparison with other annelid genomes

We compared the haplotype 1 protein set to three other annelid genomes using the orthologous and collinearity analyses of OrthoVenn3 (Sun et al. 2023), implemented with default parameters in the online module. The other genomes were the marine annelids, *Streblospio benedicti* (the closest relative with a comparable published assembly; Zakas et al. 2022) and *Capitella teleta*, and the leech *Helobdella robusta* (with the latter two genomes available in the OrthoVenn3 database). The collinearity analysis omitted *C. teleta* as only protein sequences were available for this species. Last, to compare the 11 chromosomes in our assembly with the *S. benedicti* assembly, we reciprocally BLASTed their protein sequence files using *BLAST + *v12.15.0 (Camacho et al. 2009) to generate input for a pairwise synteny search using *MCScanX* v0.8 (Wang et al. 2012). This was run with both default settings, and the number of genes required to call synteny reduced from 5 to 3. We further generated completeness metrics for both genomes (including protein sets) using *BUSCO* v5.1.3 (Simão et al. 2015).

## Results

### Raw sequencing output

PacBio HiFi sequencing produced 58.6 Gbp of reads. Summary statistics from *GenomeScope* (Supplementary Fig. S1A) estimated a genome ~707 Mb long, containing 43.7% of repetitive sequences and 4.8% heterozygosity.

### Initial scaffold-level genome assembly

The initial scaffold-level assembly was 904 Mbp long (N50 = 24 Mb), with both haplotypes showing high contiguity in chromatin contact maps (Supplementary Fig.S1B). Haplotype 1 had 583 scaffolds with a total length of 803.5 Mb, largest contig of 33.0 Mb (contig N50 = 11.7 Mb), largest scaffold of 90.0 Mb (scaffold N50 = 76.5 Mb), and 95.3% *BUSCO* completeness. Haplotype 2 had 289 scaffolds with a total length of 789.3 Mb, largest contig of 33.4 Mb (contig N50 = 11.3 Mb), largest scaffold of 92.9 Mb (scaffold N50 = 75.4 Mb), and 96.1% *BUSCO* completeness. Total *k*-mer completeness was 99.4%. See Table 2 for full assembly statistics.

**Table 2.** Sequencing and assembly statistics calculated with *QUAST* v5 and *BUSCO* v5.1.3 querying against the eukaryote database (*eukaryota_odb10*, created January 2024 with 70 genomes and 255 BUSCOs).

	Haplotype 1 assembly (scaffold-level)	Haplotype 1 assembly (chromosome-level)	Haplotype 2 assembly (scaffold-level)	Haplotype 2 assembly (chromosome-level)
Number of scaffolds	583	11	289	11
Number of contigs	676	-	381	-
Total length (bp)	803,490,011	792,440,614	789,320,953	759,533,915
Largest scaffold (bp)	89,960,646	92,870,682	92,870,682	89,960,646
Largest contig (bp)	33,040,180	-	33,497,296	-
Number of Ns per 100 kbp	2.61	2.52	2.84	2.9
Scaffold N50 (bp)	76,458,570	76,458,570	75,412,870	75,412,870
Scaffold L50	5	5	5	5
Contig N50 (bp)	11,706,541	-	11,345,269	-
Contig L50	22	-	22	-
auN	70,536,478	74,107,031	71,257,419	71,342,592
Scaffold N90 (bp)	55,310,200	55,531,921	52,145,674	52,145,674
Scaffold L90	10	10	10	10
Contig N90 (bp)	2,789,309	-	3,633,209	-
Contig L90	73	-	72	-
GC (%)	34.53%	34.39%	34.48%	34.4%
Assembly BUSCO scores				
Core genes queried	255	255	255	255
Complete BUSCOs (C)	95.3%	96.1%	96.1%	95.3%
Single-copy BUSCOs (S)	94.3%	95.7%	94.8%	95.3%
Duplicated BUSCOs (D)	0.4%	0.4%	1.2%	0.0%
Fragmented BUSCOs (F)	4.3%	3.5%	3.5%	4.3%
Missing BUSCOs (M)	0.4%	0.4%	0.4%	0.4%
Annotation stats				
Number of predicted proteins	-	30,495	-	27,423
Number of genes with at least one functional annotation	-	23,572	-	22,459
Annotation BUSCO scores				
Core genes queried	-	255	-	255
Complete BUSCOs (C)	-	93.0%	-	94.5%
Single-copy BUSCOs (S)	-	92.2%	-	94.1%
Duplicated BUSCOs (D)	-	0.8%	-	0.4%
Fragmented BUSCOs (F)	-	5.9%	-	3.9%
Missing BUSCOs (M)	-	1.1%	-	1.6%

### Chromosome-level genome assembly

Chromatin contact maps showed scaffolds to be assembled into 11 major bins, supporting the arrangement of the nuclear genome into 11 major chromosomes (Supplementary Fig. S1B). *K*-means clustering of scaffold lengths reiterated this arrangement by clustering scaffolds 1-11 together (Supplementary Fig. S2). Both the contact maps and dot-plot alignment of haplotypes (Supplementary Fig. S1B and C) identified three potential inversions: a large inversion on chromosome 2 and two smaller inversions on chromosomes 9 and 11. At this level, haplotype 1 was 792.4 Mb long with 96.1% *BUSCO* completeness, and haplotype 2 was 759.3 Mb long with 95.3% *BUSCO* completeness. Total *k*-mer completeness was 99.3%. Full assembly statistics are again in Table 2.

**Fig. 1.**
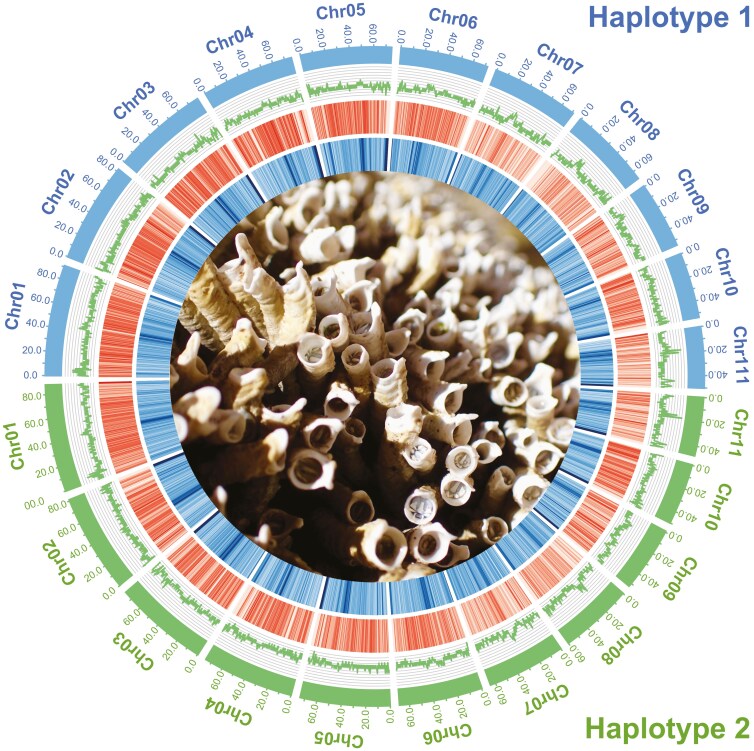
Circos plot of the 11 chromosomes in the phased assembly. From outside to inside: chromosomes of haplotype 1 (blue) and haplotype 2 (green), GC%, gene density (red), repeat density (blue), and *Galeolaria*  *caespitosa* adults retracted into tubes at low tide (photo credit: E. Chirgwin). GC%, gene density, and repeat density were estimated in 500 Kb windows with a 100 Kb step.

We softmasked ~48% of each haplotype (~379 Mbp of haplotype 1 and ~362 Mbp of haplotype 2), then characterized their repeat types and densities (Fig. 1, Supplementary Table S1). A large proportion (~36.5%) of repetitive sequences were unclassified, consistent with Zakas et al. (2022). Of those classified, most (8.1% of haplotype 1 and 8.6% of haplotype 2) were long terminal repeats.

### Mitochondrial genome assembly

Using *MITOS*, the mitochondrial genome was assembled into a circular contig 22,809 bp long, containing 38 genes (Supplementary Table S2). Of these genes, 11 coded proteins, 26 coded transfer RNAs (tRNAs), and 1 coded *12S* ribosomal RNA (rRNA). We retrieved all common protein-coding genes except *nd3* and *atp8*, and all common tRNA genes (including single and double tRNA genes and 5 copies of the *tRNA-Met* gene), but not the *16S* rRNA gene (*rrnL*).

**Fig. 2.**
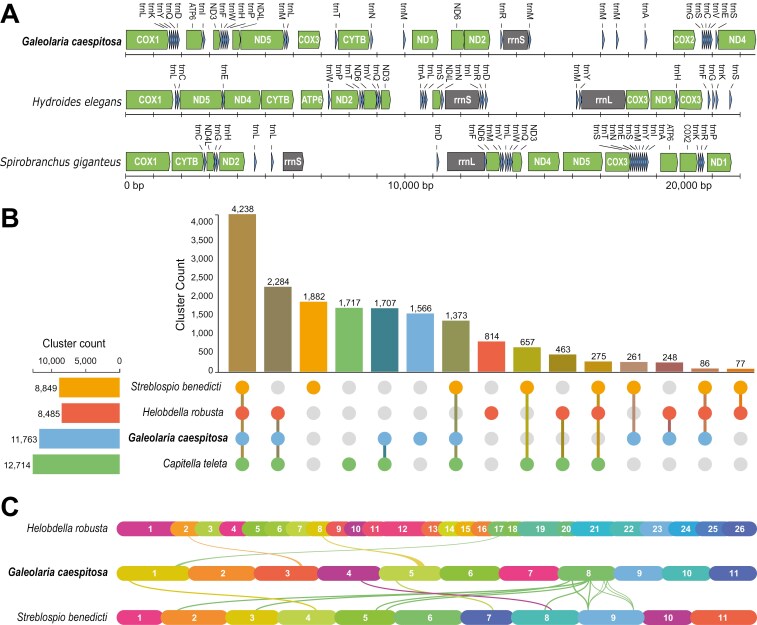
Comparative analyses of annelid genomes. (B) Counts of orthologous gene clusters shared between, *Streblospio benedicti, Helobdella robusta*, and *Capitella teleta*. (C) Collinearity analysis of *Helobdella robusta, Streblospio benedicti*, and *Galeolaria caespitosa* genomes. Note that no synteny was detected between genomes of *H. robusta* and *S. benedicti*. Genomes are labelled by chromosome number except in the case of *H. robusta*, which is labelled by scaffold.

Using *MitoFinder*, we identified *nd3*, but not 6 other protein-coding genes (*cox2*, *nd2*, *nd4*, *nd6*, and *atp6*, and *atp8*). We also identified 5 copies of the *tRNA-Met* gene, 4 copies of the *tRNA-Glu* gene, and 2 copies of the *tRNA-Gly* gene, but not the rRNA genes. We therefore completed the mitochondrial genome by extracting the gene sequence for *nd3* from the *MitoFinder* GenBank file, identifying *nd3’*s position in the *MITOS fasta* file using a *BLASTn* search, and adding *nd3* with its position to the *MITOS gff* file. This produced a final circular genome with 39 fully annotated genes. Aligning mitochondrial genomes of *G. caespitosa*, *H. elegans*, and *S. giganteus* revealed substantial gene rearrangements across species (Fig. 2A), but also failed to detect *atp8* in the latter two species despite its recovery in detailed searches by Sun et al. (2021).

### Transcriptome sequencing and genome annotation

Long-read transcriptome sequencing yielded 508,317 reads with a total length of 917 Mbp (N50 = 2,139 bp), maximum length of 12,591 bp, and 37.6% GC content. Short-read transcriptome sequencing yielded more than 20 million reads for each of the 91 embryo samples, apart from one sample with ~18.5 million. For haplotype 1, we annotated 30,495 genes, of which 23,572 (77.3%) had at least one functional annotation (Table 2, Fig. 1). For haplotype 2, we annotated 27,423 genes, of which 22,459 (81.9%) had at least one functional annotation. Our final annotated protein set had 93.0% *BUSCO* completeness for the haplotype 1 protein *fasta* file, and 94.5% for the haplotype 2 file.

### Comparison with other annelid genomes

The orthologous analysis counted 11,763 clusters in the haplotype 1 protein set. Of these clusters, 1,566 (13.3%) were unique to *G. caespitosa* and 4,238 (36.0%) were shared with all of the other annelid genomes (Fig. 2B). The collinearity analysis detected little collinearity between genomes of *G. caespitosa*, *H. robusta*, and *S. benedicti* (and none between *S. benedicti* and that of *H. robusta*; Fig. 2C). Nor did our pairwise synteny search between genomes of *G. caespitosa* and *S. benedicti*, detecting only 153 matches using default settings. Reducing the number of genes required to call synteny detected more matches (1,618), but still little synteny (Supplementary Fig. S3). The *G. caespitosa* genome had higher *BUSCO* completeness for both its assembly (95.3% *vs* 90.5% for *S. benedicti*) and annotation (93.0% *vs* 61.2% for *S. benedicti*), raising the possibility that unannotated genes in *S. benedicti* reduced synteny detection. Assembly statistics for both genomes are in Supplementary Table S3.

## Discussion

To our knowledge, this is the first phased, chromosome-level, functionally annotated reference genome (including nuclear and mitochondrial genomes) for any annelid. Phased assemblies like ours are emerging as key assets for genomic studies of marine invertebrates, especially broadcast spawners like *G. caespitosa*, which pose unique challenges due to their high heterozygosity and complex life histories (Plough 2016; Lotterhos et al. 2021; Takeuchi et al. 2022). Heterozygosity in the *G. caespitosa* genome exceeds levels reported for other annelids (Martín-Durán et al. 2021; Zakas et al. 2022). It also exceeds levels reported for broadcast spawners in other metazoan lineages, including the pearl oyster *Pinctada fucata* (Takeuchi et al. 2022), oysters in the genus *Crassostrea* (Qi et al. 2023), and the sea star *Pisaster brevispinus* (DeBiasse et al. 2022). By capturing sequence diversity between homologous chromosomes, phased assemblies offer new insights into the evolutionary origins and dynamics of such diversity across these ecologically important lineages. As such, genomic technologies that enable phasing may be essential for unlocking the full potential of marine invertebrate genomes (Takeuchi et al. 2022) and informing conservation and management strategies for rapidly changing marine ecosystems (Lopez et al. 2019).

Two lines of evidence (chromatin contact maps and *k*-means clustering of scaffolds) organize the nuclear genome of *G. caespitosa* into 11 chromosomes (2n = 22). This broadly agrees with karyotype analyses in the Serpulidae (Dasgupta and Austin 1960; Dixon et al. 1998), reporting chromosome numbers of 2n = 14 to 28 across the family and 2n = 24 to 26 for *Galeolaria*’s closest relatives (*Ficopomatus* and *Spirobranchus*) in recent phylogenies (Kupriyanova et al. 2023). The reduced number in *G. caespitosa* could reflect imprecision in inferring chromosomes from sequencing rather than karyotyping (Yamaguchi et al. 2021). Alternatively, variation in chromosome number among serpulids has been attributed to polyploidy and chromosome loss during evolution (Dasgupta and Austin 1960), and coincides with shifts in major life-history traits, including hermaphroditism (Kupriyanova et al. 2001). We saw no sign of heteromorphic sex chromosomes in *G. caespitosa*, and hermaphroditism is sequential in its closest relatives (Dixon 1981; Dixon et al. 1998) but simultaneous in more distant ones (Kupriyanova et al. 2001). New chromosome-level genomes may resolve the genetic basis of this diversity, also highlighted by our comparisons of *G. caespitosa* with *S. benedicti*, *C. teleta,* and *H. robusta*. That these genomes show little collinearity, despite sharing many orthologous clusters, suggests extensive chromosomal rearrangements among them. Limited synteny between *G. caespitosa* (order Sabellida) and *S. benedicti* (order Spionida), whose genomes have similar lengths and chromosome numbers but encode distinct life histories, may likewise support such rearrangements across the sister groups they represent (Andrade et al. 2015). Other comparisons of annelid nuclear genomes report low synteny across more distant groups (Zakas et al. 2022; Lewin et al. 2024; Schultz et al. 2024), reiterating the need for more genomes to better scrutinize these patterns.

Mitochondrial genomes are more numerous than nuclear ones, given the ease with which they are now assembled (Allio et al. 2020). The mitochondrial genome of *G. caespitosa* is similar in length to those of other serpulids, which are in turn longer than reported for other annelids (Sun et al. 2021; Struck et al. 2023). We annotated most of the 37 genes typically found in mitochondrial DNA across the Metazoa (Gissi et al. 2008; Shtolz and Mishmar 2023), apart from genes for the *16S* rRNA subunit and the protein *atp8*. Failure to detect these genes in *G. caespitosa* could reflect their fast rates of sequence evolution, consistent with their variable detection in a recent comparative analysis of other serpulids (Sun et al. 2021). Indeed, *atp8* is among the shortest, fastest-evolving mitochondrial genes, and has been lost in several metazoan lineages (Shtolz and Mishmar 2023), or erroneously reported as missing because it is highly diverged. In *G. caespitosa*’s close relatives, *S. giganteus* and *H. elegans*, for example, *atp8* was undetected by automated gene annotation (or our comparisons of mitochondrial genomes here), but was putatively recovered by highly sensitive manual searches at the protein level (Sun et al. 2021). Our comparisons also highlight gene order variation among genomes, consistent with other comparisons flagging serpulid genomes as unusually diverse in this respect (Sun et al. 2021; Struck et al. 2023). The loss or rapid divergence of mitochondrial genes in metazoans raises intriguing questions about the evolutionary drivers and functional implications that are yet to be resolved.

In summary, the high-quality genome assembled here for *G. caespitosa* will significantly enhance the genomic resources currently available for marine invertebrates. Such resources are especially valuable for annelids, which are underrepresented in comparative genomic analyses (Zakas et al. 2022; Shtolz and Mishmar 2023), but may deliver major insights into genome and life-history evolution in an exceptionally diverse group (Zakas et al. 2022; Martín-Zamora et al. 2023) and across metazoans more broadly (Paps et al. 2023). The *G. caespitosa* genome will also facilitate population genomic studies of these ecosystem engineers, whose reef-like masses of calcareous tubes enhance biodiversity on rocky shores of temperate Australia (Cole et al. 2018; Montefalcone et al. 2022). Across its range in Australia, *G. caespitosa* has diverged from its sister species (*Galeolaria gemineoa*) in a marine biodiversity hotspot warming faster than the global average rate (Hobday and Pecl 2014; Costello 2023; Gallegos et al. 2023). This system offers a new scope to study climate-driven adaptation and diversification, and the contributions of nuclear and mitochondrial genomes (Gallegos et al. 2024), in a region of high endemicity vulnerable to climate change. Doing so could highlight the transformative potential of genomic tools for advancing biodiversity research and conservation efforts.

## Supplementary Material

esaf025_suppl_Supplementary_Materials_1

## Data Availability

This Whole Genome Shotgun project has been deposited at DDBJ/ENA/GenBank under the accession JBIPTA000000000 and JBIPTB000000000. Data generated for this study are available under NCBI BioProject PRJNA1116755. Raw sequencing data for the genome assembly (NCBI BioSample SAMN41577001) are deposited in the NCBI Short Read Archive (SRA) under SRR29259014 for PacBio HiFi sequencing data and SRR29259015 for Omni-C Illumina short-read sequencing data. Raw sequencing data for the genome annotation (NCBI BioSamples SAMN43151944 for the long reads and SAMN43151945 for the short reads) are deposited on the NCBI SRA under SRR30505187 and SRR30505186 for the long and short reads, respectively. GenBank accessions for both haplotype 1 and haplotype 2 are PRJNA1116755 and PRJNA1119221. The mitochondrial genome is included in haplotype 1 (PRJNA1116755). Assembly scripts and other data for the analyses presented can be found at the following GitHub repository: https://github.com/moniquevdor/GaleolariaReferenceGenome.
